# “You Say Cognitive, I Say Cognitive”: Can Misinformation‐Informed Interventions Help Reduce Risk for Disordered Eating in Youth?

**DOI:** 10.1002/eat.24552

**Published:** 2025-10-03

**Authors:** Neophytos Georgiou, Mia L. Pellizzer, Ryan P. Balzan, Tracey D. Wade

**Affiliations:** ^1^ Flinders Institute for Mental Health and Wellbeing Flinders University Adelaide Australia; ^2^ College of Education, Psychology and Social Work Flinders University Adelaide Australia

**Keywords:** cognitive mechanisms, digital environments, eating disorders, intervention, misinformation

## Abstract

**Objective:**

This paper explores how cognitive models from misinformation research can enhance existing interventions for eating disorder (ED) risk, particularly in youth. We argue that frameworks developed to counter belief formation in misinformation offer a novel and underexplored avenue for intervening earlier in the pathway to disordered eating, particularly in environments saturated with persuasive –appearance‐ and –diet‐related content that increase ED risk.

**Method:**

We suggest that cognitive mechanisms implicated in both ED vulnerability and susceptibility to misinformation offer overlapping targets for intervention. Drawing on both literatures, we outline how –misinformation‐informed strategies such as prebunking, inoculation, and content evaluation tasks can serve as complementary, brief, digitally delivered interventions.

**Results:**

The integration of interventions tackling processing increasing ED risk with misinformation‐informed approaches may be well‐suited to reduce ED risk in young people who primarily use social media for appearance reasons. These may be more effective when placed within short‐form, algorithm‐driven social media environments where individuals encounter problematic content with limited clinical oversight.

**Discussion:**

Misinformation‐informed strategies offer new cognitive leverage points that complement existing ED interventions. When adapted thoughtfully, these tools may serve as low‐burden, scalable prevention approaches that extend support beyond the clinic and into the digital spaces where risk often emerges. We propose five concrete steps to explore this research stream.

**Summary**

- This paper proposes integrating misinformation‐informed techniques like prebunking and inoculation into single‐session interventions (SSIs) to reduce disordered eating risk online.
- It addresses a critical gap in prevention by targeting cognitive vulnerability to appearance‐related misinformation on algorithm‐driven social media.
- Practical strategies are outlined for adapting these techniques within digital eating disorder prevention, with attention to implementation barriers.
- The approach offers insights for enhancing digital literacy, content moderation, and the ethical design of online mental health tools.

## 1 Introduction

### 1.1 Social Media and Risk for an Eating Disorder

On one hand, social media offers young people opportunities for connection, self‐expression, and learning (Carah et al. 2025). Additionally, it is viewed as a “potentially viable intervention platform for offering support to persons with mental disorders, promoting engagement and retention in care, and enhancing existing mental health services” (Naslund et al. 2020, 245). On the other hand, 11% of adolescents show signs of problematic social media behaviors and experience negative mental health consequences (Boniel‐Nissim et al. 2024). There are also concerns that social media harms youth via social comparisons, cybervictimization, and fears of missing out (Carah et al. 2025). Social media often promotes appearance‐focused imagery, diet culture messaging, and health misinformation. Engagement with these platforms for appearance or body image reasons is linked to higher body dissatisfaction (Cruwys, Platow, et al. 2016; Cruwys, Leverington, et al. 2016; Dane and Bhatia 2023; de Valle et al. 2021; Georgiou et al. 2025; Rowlands et al. 2022). This exposure interacts with and exacerbates existing risk factors for eating disorder development, such as being female, dieting and/or overeating, and poor self‐worth (Fittig and Jacobi 2024; Wade 2025).

Concern about the harms of social media has resulted in government policies regulating use in young people: the United States requires children to be 13 years or older to create new social media accounts, France requires that social media platforms refuse access to children under 15 unless they have parental permission, and Australia has introduced a law to ban children under 16 from accessing social media platforms. While young people do not want to be excluded from the digital world, they express a desire for practical education on responsible phone use related to social media (Bar et al. 2025). A study of young people who self‐reported that the primary reasons for their social media use were appearance‐related suggested that an intervention helping them curate their social media to make it less harmful had few benefits, apart from a reduction in appearance comparison (de Valle and Wade 2022). In contrast, an intervention targeting self‐criticism showed a greater impact on reducing appearance comparison while producing small to moderate decreases in the use of social media for appearance motivations and improvements in body image (de Valle and Wade 2022). Disordered eating was not impacted by either intervention. This finding suggests that targeting processes relevant to disordered eating is a promising avenue for decreasing harmful appearance‐motivated social media use, but such interventions could be further strengthened. This Spotlight article explores the rationale for integrating interventions that target processes such as self‐criticism, which increase risk for disordered eating, with those suggested by misinformation models. Together, these approaches aim to strengthen our ability to teach at‐risk youth helpful social media use and reduce the risk of developing disordered eating.

### 1.2 Utilizing Misinformation Models in Our Social Media Interventions

In online environments, misleading information often spreads quickly and is rarely scrutinized before being shared (Lewandowsky et al. 2017; Murphy et al. 2023). For instance, TikTok, the fastest‐growing social media platform among children and young people, has drawn nearly 44 billion views on videos tagged #mentalhealth (Motta et al. 2024). Notably, up to a third of these videos promote eating disorders and receive similar levels of engagement as accurate, recovery‐focused content (Lookingbill et al. 2023). The algorithmic design of these platforms reinforces the problem, as even small signs of engagement with a particular topic—including pro–eating disorder content—prompt the system to recommend similar material repeatedly, creating a cycle that sustains and normalizes harmful content (Griffiths et al. 2024).

To address this cycle and false content more broadly, misinformation prevention models focus on three sequential aspects of how people engage with online content: (a) *attention*, or what aspects they show preference for when viewing online material (Pennycook and Rand 2019), (b) *interpretation*, or how they make sense of the material (e.g., what the content means to them; Pennycook et al. 2018; Lewandowsky et al. 2017), and (c) *belief updating*, or how exposure to misinformation changes the way a person views themselves or beliefs about their world/environment (Ecker et al. 2017; Pennycook et al. 2021; see Table 1).

**TABLE 1:** Key definitions and forms of misinformation interventions.

Intervention type	Description	Key features/techniques	Example formats
Inoculation	A psychological framework, first developed in persuasion research, that builds resistance to misinformation by providing counterarguments in advance.	Inoculation involves two essential components: forewarning that misleading information will be encountered and a refutation that explains why the claim is inaccurate or manipulative. These elements create cognitive resistance to persuasion.	Classroom‐based exercises where students are exposed to weakened misinformation and guided through refutations (McGuire 1964); digital inoculation campaigns on social media that present common misinformation strategies alongside counterarguments (Lewandowsky and van der Linden 2021).
Prebunking (“Attitudinal Inoculation”)	A pre‐emptive application of inoculation theory that seeks to reduce the impact of misinformation before exposure.	Prebunking combines a warning that misinformation will be encountered, an explanation of how it is misleading, and a weakened or “microdosed” example that enables later recognition of similar tactics.	Short explanatory videos that walk viewers through common manipulation strategies (Roozenbeek et al. 2020); infographics that visually illustrate tactics (UNESCO 2020); interactive online games such as *Bad News* and *Go Viral!* where players adopt the role of a misinformation creator to learn how persuasive tactics operate (Roozenbeek and van der Linden 2019; Basol et al. 2020).
Debunking inoculation	An application of inoculation delivered after limited exposure but before misinformation becomes fully persuasive.	Debunking inoculation provides corrective information while also strengthening resistance to future misinformation by embedding refutations.	Fact‐checks that not only correct misinformation but also highlight the manipulative strategies used (Ecker et al. 2017).
“Forewarning” prebunking	Alerts individuals that they are about to encounter misleading or manipulative content.	Forewarning heightens critical scrutiny and activates psychological defenses, encouraging audiences to evaluate the credibility of incoming material.	Pop‐up or banner warnings presented immediately before online advertisements or social media posts to cue critical evaluation (Roozenbeek et al. 2020).
“Pre‐Emptive Refutation”	Offers rebuttals to misinformation before individuals encounter it organically.	By pairing a weakened example of misinformation with an immediate refutation, pre‐emptive refutation equips individuals to dismiss similar arguments when they later arise.	Climate denial prebunking delivered through short videos that first present a weak false claim and then explain why it is incorrect (Harjani et al. 2022).
“Post‐Exposure Correction”	A corrective strategy delivered after misinformation has been encountered.	Post‐exposure correction identifies the false claim, provides accurate information, and explains why the claim is misleading. Its effectiveness depends on timing, repetition, and the perceived credibility of the source.	Fact‐checking websites that provide detailed refutations (Pennycook et al. 2021) and peer‐to‐peer corrections where individuals challenge false claims in conversation (Ecker et al. 2022).
“Reframing”	A communication strategy that alters how information is presented to weaken the persuasive pull of misinformation.	Reframing highlights manipulative tactics or shifts attention to alternative values and explanations, thereby reducing the influence of misleading narratives.	Scientific consensus messaging in climate communication that highlights widespread agreement among experts to undercut misinformation (Lewandowsky et al. 2017).
Accuracy nudges	Simple prompts that encourage individuals to consider the accuracy of content before sharing or endorsing it.	Accuracy nudges work by shifting attention toward truthfulness as a decision‐making criterion, reducing impulsive sharing of misinformation without requiring explicit correction.	Social media prompts that explicitly ask users to reflect on content accuracy before sharing, e.g., “Is this information accurate?” (Pennycook et al. 2021).
Fact‐checking and labeling	Corrective approaches that directly signal whether information is true or false, often accompanied by credibility labels.	Fact‐checking works by explicitly flagging false content and presenting the correct information, while labeling interventions add visual indicators of reliability. Their impact depends on the perceived trust in the fact‐checker.	Professional fact‐checking sites that provide detailed evidence‐based assessments (e.g., Snopes, PolitiFact), or warning labels such as “False Information” tags used on Meta platforms (Walter and Murphy 2018).

*Note*: Adapted from Harjani et al. (2022), and supplemented with key studies on inoculation, prebunking, and correction.

Evidence from recent research illustrates the relevance of these processes to the context of eating disorders, showing that individuals at risk are particularly susceptible to both diet‐related and general misinformation. Studies highlight parallels across the three domains: in terms of *attention*, at‐risk individuals show heightened focus on weight and shape cues; in terms of *interpretation*, they often construe appearance‐ or health‐related messages in rigid and self‐critical ways; and in terms of *belief updating*, repeated exposure can strengthen thin‐ideal beliefs and reinforce distorted self‐perceptions. These cognitive patterns are further compounded by social identity processes, such as seeking belonging through group norms around dieting and body image (e.g., the “Pro‐Ana” movement; Cruwys, Platow, et al. 2016; Cruwys, Leverington, et al. 2016; Georgiou et al. 2025). These findings suggest misinformation models are directly relevant to understanding vulnerability in eating disorders.

### 1.3 Our Novel Research Question

Moving forward, we propose that models from misinformation research be evaluated for their relevance to social media content that elevates eating disorder risk. Additionally, the use of strategies developed within misinformation research should be considered. These interventions, described in Table 1, aim to build resistance by training individuals to evaluate whether a source is trustworthy and to adopt more flexible ways of thinking about online content. Finally, given the high level of ambivalence about seeking help for an eating disorder, which contributes to low rates of help‐seeking (Radunz et al. 2023), we suggest that these approaches be embedded within interventions targeting underlying risk processes, such as self‐criticism (Paranjothy and Wade 2024). Framing interventions around these broader processes may be more engaging for youth than a direct focus on disordered eating. The interventions need to be engaging and able to compete with popular platforms of “short‐form video content” that are designed for rapid consumption, high engagement, and algorithmic distribution across social media platforms (Jain et al. 2025).

### 1.4 Cognitive Obstacles (for Researchers)

Despite a shared interest in cognitive mechanisms, research related to misinformation and eating disorder mechanisms operates on fundamentally different assumptions about how beliefs are formed and how they change. Misinformation studies typically view beliefs as fast‐forming, malleable, and responsive to timely interventions, especially in dynamic digital spaces. These strategies are typically brief, low‐cost, and designed to build resistance by helping individuals detect persuasive manipulation and reject misleading content (Lewandowsky and van der Linden 2021; Pennycook and Rand 2019). They rest on specific assumptions: that beliefs are modifiable, that change can occur through short exposure, and that timing and sequencing matter.

The beliefs that underpin the mechanisms or processes that underlie eating disorder risk, for example, self‐criticism, self‐worth, perfectionism (Wade et al. 2025), are rarely formed in a single moment; they emerge gradually over the lifetime and are reinforced over time through emotional learning, experience, and ongoing selective attention bias. They can be experienced as protective, deeply stabilizing, and tied to identity.

The challenge is not whether misinformation models are relevant, but how they can be integrated and applied into interventions that seek to help youth develop skills for managing social media content, such that it maximizes benefits and reduces harm. Cognitive tools from misinformation research should be seen not as replacements, but as complementary assets—offering new leverage points for intervention when adapted with careful attention to what kinds of belief structures they are best suited to address.

### 1.5 Concrete Steps

The challenge of integrating misinformation models with interventions for eating disorder risk is not insurmountable. Given the high profile of, and concern about, social media use in youth, we suggest the development of digital interventions that integrate: (1) an explicit focus on healthy social media use in young people, (2) brief psychoeducation as to the factors that make young people more susceptible to harm, stressing the relevance of both long‐term risk factors and misinformation “in the moment,” (3) targeting of transdiagnostic cognitive behavioral processes that influence disordered eating, depression, and anxiety (Wade et al. 2025) in a brief and engaging manner that maximizes change (Schleider et al. 2020), and (4) “doing” exercises related to managing social media misinformation, informed by the interventions presented in Table 1. Importantly, the groundwork for such approaches already exists. Cheung et al. (2025) found that users perceive digital eating disorder tools as practical and effective—but they also emphasize the importance of *personalization*, user cocreation, and positioning these tools within *stepped‐care frameworks*, with flexibility around professional versus peer support. Digital eating disorder tools include online CBT modules, self‐guided apps, and chatbot‐delivered supports (e.g., Aardoom et al. 2013; Duggan et al. 2025; Sharp et al. 2025; Wilksch et al. 2025). A potential approach would be to incorporate misinformation‐informed strategies to existing tools and adapt delivery to short‐form social media contexts (Balzan et al. 2023; Thompson et al. 2025). Clinically, these approaches show promise as early, low‐burden prevention tools that reach individuals before symptom escalation—even those not yet seeking formal treatment.

In Table 2, we outline various concrete steps toward addressing this knowledge gap. The first recommendation is to use a dismantling study to establish the relative usefulness of a single session intervention (SSI) targeting processes increasing risk for disordered eating (e.g., self‐criticism, cognitive inflexibility) to a misinformation‐informed SSI and a combined approach. Our second recommendation is to develop digital interventions that align with how young people use social media, are perceived as convincing sources of support, and appear at the point of need within their social media feed. Saltz et al. (2021) highlight that misinformation‐based interventions for adolescent populations risk being dismissed as biased or paternalistic if they are not designed with user perspectives in mind via co‐design and iterative testing. Moreover, Liu et al. (2024) shows that corrective misinformation interventions (e.g., *prebunking* or *debunking* inoculation; see Table 1) are received more positively when they come from familiar sources (e.g., peers, friends), suggesting that interventions should focus on trust‐building and integration within peer networks rather than appearing as an institutional authority (e.g., government health departments or international expert bodies such as the World Health Organization; Ecker et al. 2022). The effectiveness of such intervention efforts can also be improved by strategic positioning, using established techniques such as forewarning, preemptive refutation, and postexposure correction (see Table 1 for details; Walter and Murphy 2018; Ecker et al. 2017).

**TABLE 2:** Concrete steps for integrating misinformation‐informed interventions into eating disorder prevention.

Future recommendations	Action
Rapidly test combined approaches in single session intervention(s)	Conduct a four‐arm randomized controlled trial comparing: (a) waitlist, (b) an SSI targeting eating disorder–relevant cognitive mechanisms (e.g., self‐criticism, cognitive inflexibility), (c) a misinformation‐informed SSI (e.g., prebunking or inoculation), and (d) a combined approach. This design would allow assessment of additive or interactive effects on eating disorder risk factors.
2 Apply specifically to short‐form content users	Focus on high‐use short‐form content consumers (e.g., TikTok users aged 14–25) by tailoring stimuli to match platform‐specific features such as persuasive design, pacing, and tone. Pre‐screen participants for baseline eating disorder risk and patterns of exposure to appearance‐ or diet‐related content.
3 Use belief‐ and ED‐relevant outcome measures	Include outcome measures that capture both eating disorder pathology (e.g., weight concern, dietary restraint) and misinformation‐relevant cognitive processes (e.g., source scrutiny, and resistance to persuasive content).
4 Leverage existing digital ED platforms	Partner with platforms already delivering ED‐focused content (e.g., mobile apps, browser extensions). Integrate misinformation modules as add‐ons or “pre‐session tasks” to test user acceptability and effectiveness.
5 Address implementation risks and engagement	Pilot test interventions for timing, tone, and credibility using experimental A/B testing designs. Collect qualitative feedback from at‐risk users on potential barriers such as lack of trust, perceptions of co‐optation, or disengagement. Use these insights to refine delivery and narrative framing iteratively.

Our final three recommendations relate to testing such interventions, in terms of measures to use, platforms on which to host such interventions, and examination of implementation risks. This program of research can inform whether we can further equip young people to use social media in such a way as to minimize harm and maximize benefit.

## 2 Conclusions: The “Other 23 h” in a Day

People aged between 13 and 18 years have upwards of 8.5 h of daily screen time (Nagata et al. 2024). To complement existing interventions, and for those who have not sought treatment, there is a growing need for online tools that engage individuals during the “other 23 h of the day” which are impacted by what individuals see, share, and internalize from online content.

Cognitive approaches already exist within eating disorder interventions that support the use of techniques to combat media messaging, such as the use of cognitive dissonance for the thin‐ideal (e.g., Stice et al. 2019, 2020, 2021), and media literacy that supports youth to become critical consumers of media (e.g., Wilksch et al. 2025). Insights from misinformation research offer a distinct and complementary perspective tested extensively in the context of the rapidly developing online world that youth inhabit. The question of whether this is a more effective approach in helping combat the harm of social media is another empirical question that future research needs to address.

Ultimately, misinformation interventions represent strategies for reducing the persuasive pull of the *message* itself (e.g., reframing or inoculation against harmful social media content) and considering the role of the *messenger* (e.g., whether information comes from peers, influencers, or institutions). This perspective complements existing interventions for reducing risk for disordered eating, which primarily focus on characteristics of the *recipient* that influence the internalization and response to dieting or appearance‐related content.

## Author Contributions

**Neophytos Georgiou:** conceptualization, investigation, writing – original draft, writing – review and editing, resources. **Mia L. Pellizzer:** writing – original draft, writing – review and editing, investigation. **Ryan P. Balzan:** writing – original draft, writing – review and editing. **Tracey D. Wade:** writing – original draft, writing – review and editing, supervision.

## Conflicts of Interest

The authors declare no conflicts of interest.

## Data Availability

Data sharing not applicable to this article as no datasets were generated or analysed during the current study.
